# The role of customer service manual on workplace emotional burden in nationwide cross sectional study

**DOI:** 10.1186/s40557-019-0285-9

**Published:** 2019-02-12

**Authors:** Sehyun Yun, Sung-Shil Lim, Jihyun Kim, Young-Kwang Kim, Jong-Uk Won, Jin-Ha Yoon

**Affiliations:** 1 Department of Preventive Medicine, Yonsei University College of Medicine, The Institute for Occupational Health College of Medicine, 50, Yonsei-ro, Seodaemun-gu, Seoul, 120-749 South Korea; 2 Graduate School of Public Health, Yonsei University College of Medicine, Soul, South Korea; 3 Incheon Worker’s Health Center, Incheon, South Korea; 4 Department of Preventive Medicine, Yonsei University College of Medicine, Seoul, South Korea

**Keywords:** Customer service manual, Emotional burden, Emotional demand, Service industry, Mental health, Korea working condition survey

## Abstract

**Background:**

We aim to discuss the overall effect of customer service manual (CSM) on service industry workers using Korean Working Condition Survey.

**Methods:**

Out of 50,007 total survey participants, 11,946 customer service workers were included in the current study (5613 men, 6333 women). Answers to survey questions were used to define the use of CSM, emotional burden, emotional dissonance, engaging angry customers and other covariates. Emotional burden included either depressive event or stress level. Odds ratio (OR) with 95% confidence interval (95% CI) of experiencing emotional burden was calculated by logistic regression model. Interaction effect between CSM and engaging angry customer on emotional burden was also estimated.

**Results:**

Out of 11,946 subjects, total of 3279 (27.4%) have experienced emotional burden. OR (95% CI) of experiencing emotional burden was 1.40 (1.19–1.64) in men and 1.25 (1.09–1.44) in women. There was gender difference in interaction effect between the use of CSM and engaging angry customers. In men, OR (95% CI) was 3.16 (1.38–7.23) with additive effect when always engaging angry customers with CSM compared to rarely engaging without CSM, while in women OR (95% CI) was 8.85 (3.96–19.75) with synergistic effect. Moreover, the risk of depressive event increased only in women with OR (95% CI) 2.22 (1.42–3.48).

**Conclusions:**

Our current study highlighted association between emotional burden and CSM in both men and women service workers. Furthermore, women were affected more severely by CSM. The results from current study suggest that CSM should be changed appropriately to benefit workers.

## Introduction

Customer satisfaction seems to be the most highly sought value in today’s business world. Increasing number of companies, whether big or small, is focusing on and allocating more people in customer service department. According to the World Bank data from 2015, service industry accounted for 68.9% of world GDP, which is more than a 10% increase compared to that of 20 years ago. This trend is more apparent in Asian countries where recent economic development emphasized the importance of customer satisfaction. For example, in China percentage of GDP dedicated to service industry showed 17% increase in the past 20 years, Philippines 13% in the same period and Korea 5% [1]. As the size of service sector becomes larger, health issues associated with the industry, most importantly emotional labor [2], should come into a view.

Emotional labor, simply put, is a state of expressing or suppressing certain emotion required by jobs [3]. For example, employees may need to exert excessively positive attitude in greeting customers even when they are severely depressed. In other cases, companies may require workers to suppress negative feelings against customers even when customers are illogically demanding. As a result, emotional labor, although a necessary component of almost any job, can lead to absenteeism, job anxiety and general negative work performances [4]. Workers who are chronically exposed to emotional burden can also develop burnout syndrome [5, 6] which, by definition, is a combined state of emotional exhaustion and depersonalization [7]. In many cases, excessive emotional demand may result in serious psychological problems such as depression [8]. Therefore it is not too far to say that emotional labor impose highly deteriorating effect on mental health of service sector workers.

The exact mechanism of emotional labor leading to deterioration of mental health has not yet been fully investigated. One of the proposed explanations is emotional dissonance. This refers to discrepancies between the felt and displayed emotion [9, 10]. This gap is seen as a type of role conflict which is a strong indicator of depersonalization [11] and emotional exhaustion [12]. Continued exposure to emotional exhaustion can lead to emotional burnout [13] which can then cause mental illness [8, 14] and, in many cases, suicidal ideation [15].

Emotional labor is a definite threat to those constituting a continuously-increasing service industry. Mental fatigue due to concealing emotions is found to be linked to several adverse health effects such as cardiovascular disease and cancers [16]. The prevalence of health deterioration related to occupational stress is increasing across the globe [17]. Instead of addressing this issue, however, the industry is pushed to further worsen the problem for the sake of customer satisfaction. One of the major devices to improve customer satisfaction is customer service manual (CSM) which is a standardized manual with specific guidelines in expressing emotions in engaging customers. By aiding employees to accurately address specific customer needs and pleasing customers at the same time, the guide clearly benefits customer satisfaction. Its effect on employees who are required to follow the guideline, however, is disputable. CSM eliminates the need to think and make decisions for workers. Moreover, by projecting emotion indicated by the guideline, employees can distance themselves from the implicated emotion [18]. However, at the same time, CSM strictly limits the freedom of expression. Employees must regulate their feelings to display company-standard emotion which is an important job requirement [19]. This discrepancy, as explained earlier, can lead to lowered self-esteem, depression, and alienation from work [18]. Therefore, it is difficult to determine the combined effect of CSM on employee and unfortunately there are not enough study conducted on the impact of CSM on emotional demand.

Hence, this study aims to investigate the association between CSM and emotional burden on employees. A national representative data involving more than 50,000 workers were utilized to conduct a comprehensive analysis on emotional burden. The data are collected from the Korean Working Condition Survey (KWCS), which is modeled after European Working Condition Survey to monitor occupational information. Other factors, such as gender, monthly household income, job satisfaction, job type, weekly working hours that can affect emotional burden were also considered in this study.

## Materials and methods

**Fig. 1:**
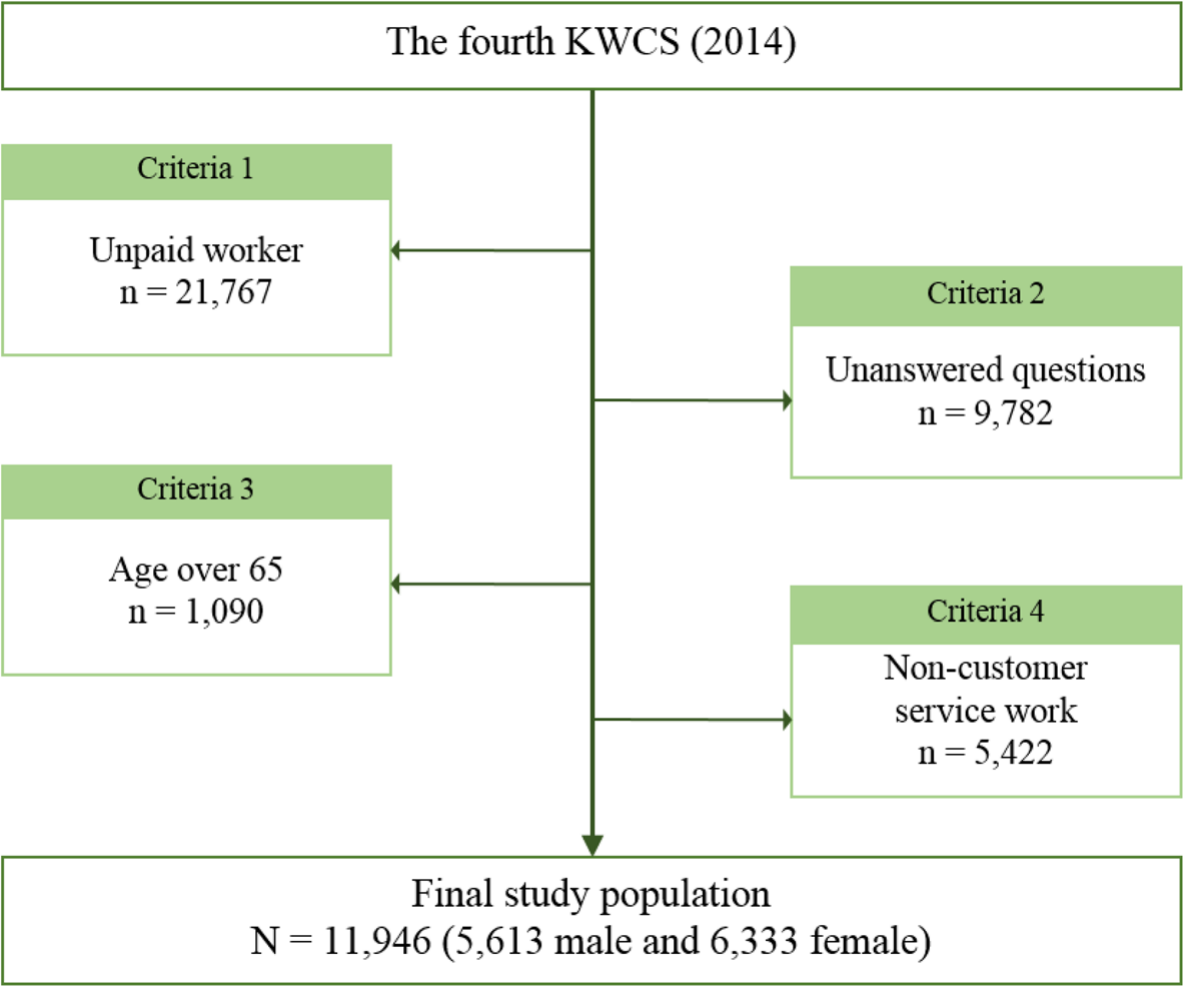
Study population selection process schematics

Data for this study were obtained from the fourth Korean Working Condition Survey (KWCS) (2014). The survey, directed by the Korea Occupational Safety and Health Agency, is similar to European Working Condition Survey with some modifications in survey questions to best fit Korean population. The KWCS includes a total of 50,007 consented working individuals over the age of 15 years whom are selected randomly to represent the actual Korean working population. The following criteria were applied to distinguish a sample that best serve the study: 1) only paid worker, 2) answered all relevant survey questions, 3) age less than 65 years, and 4) engage in customer service work. Following these criteria, unpaid workers (*n* = 21,767) were excluded to only account for paid workers. Those who did not answer all the study-relevant questions were also excluded (*n* = 9782). People over the age of 65 years (*n* = 1090) were also excluded to best simulate pre-retirement population. To distinguish those engaging in customer service work, a question “Does your job include directly dealing with individuals who are not business partners e.g. customers, passengers, students, or patients?” was considered. The possible answer choices for the question were ‘always’, ‘almost always’, ‘3/4 of working hours’, ‘half of working hours’, ‘1/4 of working hours’, ‘almost never’, and ‘never.’ People were considered not to participate in customer service work and thus excluded from the study if they responded ‘never’ or did not give an answer (*n* = 5422). After the selection process (Fig. 1), a total of 11,946 subjects (5613 men and 6333 women) were included in the final analysis.

### Emotional burden

It is difficult to objectively quantify emotional burden. Assuming that severe emotional load leads to negative mental outcome, emotional burden is estimated through stress and depressive events. Therefore, in this study, a person was considered to have experienced emotional burden if he suffered stress or depression. Participants were asked if they experienced mental stress at work or depression or anxiety disorder in the past 12 months. Those who answered “Always” or “Almost always” to the question “Select a choice that best describe your work” with a sub-question “I experience stress at work” and subjects who answered “Yes” to question “Have you experienced the following health problem in the past 12 months?” with a sub-question “Depression or anxiety disorder” were considered to have emotional burden.

### Customer service manual

Whether the participants were required to use customer service manual was determined by the question “In regard to your work, is there a company-mandatory service manual on emotional expression?” The participants were to choose between “Yes” and “No.” Those who answered “Yes” were considered to have customer service manual in their work and the others not.

### Emotional dissonance

Possible explanation of emotional burden caused by CSM is emotional dissonance [12]. To test the hypothesis, the effect of emotional dissonance on emotional burden as well as the role of CSM in creating emotional dissonance were investigated. Whether the subjects experienced the gap between felt and displayed emotion was approximated through the question “Do you have to suppress emotion at work?” Participants were separated into three categories based on degree of suppressing emotion: rarely (never and almost never), sometimes (1/4 of working hours), always (half of working hours, 3/4 of working hours, almost always, always).

### Engaging angry customers

The study subjects were also asked to answer “Does your job include dealing with upset customers or patients?” with ‘always’, ‘almost always’, ‘3/4 of working hours’, ‘half of working hours’, ‘1/4 of working hours’, ‘almost never’, ‘never’, or ‘NA.’ The answers were re-categorized into “Rarely” (NA, never, almost never), “Sometimes” (1/4 of working hours), and “Always” (half of working hours, 3/4 of working hours, almost always, always).

Each participant were then allocated into six groups in a 2 by 3 matrix of “Yes” and “No” for use of customer service manual and “Rarely”, “Sometimes” and “Always” for interacting with angry customers. According to this methodology subjects who used customer service manual and rarely deals with upset customers were separated into group 1, those who did not use customer service manual and rarely deals with upset customers into group 2 and so on to determine the interactive effect between CSM and dealing with angry customers.

### Covariates

Information on the following covariates were obtained from the fourth KWCS: age, monthly household income, education, weekly working hours, job satisfaction, job schedule, job class, and occupational classification. The covariates were categorized by answer choices on the survey questions or re-grouped to best fit the study. Age was re-categorized into five: below 25 years, between 25 and 34 years, between 35 and 44 years, between 45 and 54 years and between 55 and 64 years. Monthly household income was calculated in US dollars and separated into less than 1000 dollars, between 1000 and 1999 dollars, between 2000 and 2999 dollars, and more than or equal to 3000 dollars. Weekly working hours were subdivided into three groups: less than 40 h, between 40 and 49 h, and more than or equal to 50 h. The survey question “How do you feel about general working condition at your current job?” addressed whether the participants were satisfied with their jobs. The answer choices “very not satisfied” and “not satisfied” were categorized into not satisfied and “satisfied” and “very satisfied” were categorized into satisfied. Job schedules were either fixed schedule or shift schedule for any non-fixed schedules. Job class indicates whether the subjects were guaranteed of regular retirement or pension for retirement (permanent) or daily or temporarily employed (temporary). The ten major International Standard Classifications of occupations groups were re-grouped into three under occupational classification: office workers (managers, professionals, and technicians and associate professionals), sales and service workers (clerical support workers and service and sales worker), and manual worker (skilled agricultural, forestry and fishery workers, craft and related trades workers, plant and machine operators, assemblers and elementary occupations). Finally, the time spent managing customers were separated into three: “Rarely” (almost never), “Sometimes” (1/4 of working hours) and “Always” (half of working hours, 3/4 of working hours, almost always, always).

### Statistical analysis

Chi-square tests were used to compare socioeconomic characteristics and occupational status based on the presence of emotional demand (Table 1). The odds ratio (OR) and 95% confidence intervals (95% CIs) on the presence of emotional demand were calculated using a fully adjusted multiple logistic regression model. The relationship between CSM and emotional dissonance was also estimated by the OR and 95% CIs calculated by fully adjusted multiple logistic regression model. The degree of interaction between the use of customer service manual and engaging with angry customers on the presence of depressive events was estimated through *p*-value using a logistic regression model. A *p*-value less than 0.05 was considered statistically significant.

**Table 1:** Basic characteristics of study population based on emotional burden and gender

Parameters	Emotional Burden, No. (%)					
	Men (*n* = 5613)			Women (*n* = 6333)		
	Yes	No	*p*-value	Yes	No	*p*-value
Total	1562 (27.8)	4051 (72.2)		1717 (27.0)	4653 (73.1)	
Age			0.008			0.013
< 25	80 (23.2)	265 (76.8)		105 (23.7)	338 (76.3)	
25–34	359 (27.5)	948 (72.5)		394 (28.5)	988 (71.5)	
35–44	481 (28.2)	1222 (71.8)		479 (26.7)	1315 (73.3)	
45–54	430 (30.7)	970 (69.3)		534 (27.8)	1386 (72.2)	
55–64	212 (24.7)	646 (75.3)		205 (24.7)	626 (75.3)	
Monthly household income, USD			< 0.001			< 0.001
< 1000	53 (18.8)	229 (81.2)		145 (17.6)	678 (82.4)	
1000 - 1999	309 (24.5)	955 (75.6)		949 (27.8)	2468 (72.2)	
2000 - 2999	531 (27.6)	1391 (72.4)		407 (27.7)	1064 (72.3)	
≥ 3000	669 (31.2)	1476 (68.8)		216 (32.8)	443 (67.2)	
Education level			0.062			< 0.001
< Middle school	17 (22.1)	60 (77.9)		20 (15.4)	110 (84.6)	
< High school	62 (22.5)	214 (77.5)		78 (20.8)	297 (79.2)	
≥ High school	1483 (28.2)	3777 (71.8)		1619 (27.6)	4246 (72.4)	
Weekly working hours			< 0.001			< 0.001
< 40	54 (16.9)	266 (83.1)		179 (21.1)	670 (78.9)	
40–49	1012 (28.6)	2529 (71.4)		1101 (27.4)	2921 (72.6)	
≥ 50	496 (28.3)	1256 (71.7)		437 (29.2)	1062 (70.8)	
Job satisfaction			< 0.001			< 0.001
Satisfied	1085 (26.1)	3079 (73.9)		1183 (24.4)	3665 (75.6)	
Unsatisfied	477 (32.9)	972 (67.1)		534 (35.1)	988 (64.9)	
Job schedule			0.018			0.034
Fixed	1354 (27.3)	3603 (72.7)		1548 (30.8)	4273 (69.2)	
Shift	208 (31.7)	448 (68.3)		169 (26.6)	380 (73.4)	
Job class			0.025			< 0.001
Permanent	1327 (28.4)	3340 (71.6)		1334 (28.2)	3404 (71.8)	
Temporary	235 (24.8)	711 (75.2)		383 (23.5)	1249 (76.5)	
Suppress emotion at worksite			< 0.001			< 0.001
Rarely	343 (26.1)	973 (73.9)		349 (25.0)	1047 (75.0)	
Sometimes	981 (26.2)	2770 (73.8)		1079 (25.3)	31,945 (74.8)	
Always	238 (43.6)	308 (56.4)		289 (41.2)	412 (58.8)	
Engaging angry customer			< 0.001			0.012
Rarely	162 (9.6)	1527 (90.4)		134 (8.0)	1534 (92.0)	
Sometimes	647 (25.3)	1908 (74.7)		606 (21.4)	2225 (78.6)	
Always	753 (55.0)	616 (45.0)		977 (52.2)	894 (47.8)	
Use of customer service manual			< 0.001			< 0.001
Yes	295 (35.9)	526 (64.1)		396 (32.1)	838 (67.9)	
No	1267 (26.4)	3525 (73.6)		1321 (25.7)	3815 (74.3)	

## Results

Table 1 shows the basic characteristic of the study population. Out of 11,946 subjects, a total of 3279 (27.4%) have experienced emotional burden. There were 5613 (47.0%) man workers with 1562 (27.8%) employees who were classified to have felt emotional burden. For 6333 woman workers, comprising 53.0% of total population, 1717 (27.0%) of them have experienced emotional burden.

Table 2 includes the association between the use of customer service manual and the presence of stress, depressive event or emotional burden, which is a combination of stress and depressive event. The ORs were adjusted for covariates such as age, monthly household income, education level, weekly working hours, job satisfaction, job schedule, job class, and occupational classification. Both man and woman workers showed increased risk of emotional burden when using customer service manual with man employees showing a higher risk (OR 1.40; 95% CI 1.19–1.64 for men and OR 1.25; 95% CI 1.09–1.44 for women). Only woman workers displayed a statistically significant increase in risk of depressive event in the presence of customer service manual (OR 2.22; 95% CI 1.42–3.48). In the case of stress, both man and woman workers presented an increase in risk with higher risk for man employees (OR 1.41; 95% CI 1.20–1.66 and OR 1.21; 95% CI 1.05–1.39 for women).

**Table 2:** The relative risk of emotional burden, stress, and depressive event based on the use of customer service manual, suppressed emotion or engaging angry customers by multiple logistic regression model

Variables	Odds ratio (95% confidence interval)	
	Men	Women
Emotional Burden (Stress or Depressive event)		
Use of customer service manual		
Yes	**1.40 (1.19–1.64)**	**1.25 (1.09–1.44)**
No	1.00 (reference)	1.00 (reference)
Stress		
Use of customer service manual		
Yes	**1.41 (1.20–1.66)**	**1.21 (1.05–1.39)**
No	1.00 (reference)	1.00 (reference)
Depressive event		
Use of customer service manual		
Yes	**1.15 (0.52–2.53)**	**2.22 (1.42–3.48)**
No	1.00 (reference)	1.00 (reference)
Suppressed emotion at worksite		
Rarely	1.00 (reference)	1.00 (reference)
Sometimes	**3.11 (2.58–3.75)**	**3.07 (2.51–3.75)**
Always	**11.05 (9.06–13.48)**	**12.26 (10.00–15.03)**
Angry customer		
Rarely	1.00 (reference)	1.00 (reference)
Sometimes	**1.65 (1.33–2.05)**	**1.73 (1.42–2.11)**
Always	**2.57 (1.82–3.65)**	**2.46 (1.84–3.28)**

Adjusted for covariates including age, monthly household income, education, weekly working hours, job satisfaction, job schedule, job class, occupational classification, **and time spent managing customers**

Relationship between customer service manual utilization and suppressing emotion is described in Table 3. For both man and woman workers, using customer service manual at work resulted in higher probability of suppressing emotion at work with minimal difference in risk between genders (OR 1.60; 95% CI 1.39–1.85 for men and OR 1.55; 95% CI 1.37–1.74 for women). As predicted, suppressing emotion at work leads to emotional burden. Furthermore, as the time spent suppressing emotion at work increases the risk of emotional burden also increases as evidenced by the Table 2. This is true for both man and woman workers (OR 3.11; 95% CI 2.58–3.75 for men who sometimes suppress emotion, OR 3.07; 95% CI 2.51–3.75 for women & sometimes, OR 11.05; 95% CI 9.06–13.48 for men & always and OR 12.26; 95% CI 10.00–15.03 for women & always).

**Table 3:** The relative risk of always suppressing emotion at work based on the use of customer service manual by multiple logistic regression model

Variables	Odds ratio (95% confidence interval)	
	Men	Women
Use of customer service manual		
Yes	**1.60 (1.39–1.85)**	**1.55 (1.37–1.74)**
No	1.00 (reference)	1.00 (reference)

Adjusted for covariates including age, monthly household income, education, weekly working hours, job satisfaction, job schedule, job class, occupational classification, **and time spent managing customers**

There is also a positive relationship between the time dealing with demanding customers and emotional burden as evidenced by Table 2. (OR 1.65; 95% CI 1.33–2.05 for men who sometimes engage demanding customers, OR 1.73; 95% CI 1.42–2.11 for women & sometimes, OR 2.57; 95% CI 1.82–3.65 for men & always and OR 2.46; 95% CI 1.84–3.28 for women & always). Interactive effect between customer service manual and dealing with demanding customers on emotional burden was analyzed. Interestingly, as described in Table 4, there was a negative effect between the two variables in man workers while synergistic effect was apparent between the same variables only in woman workers. (OR 3.16; 95% CI 1.38–7.23 for man who uses CSM and always interact with angry customers and OR 8.85; 95% CI 3.96–19.75 for woman counterparts).

**Table 4:** The interaction effect between customer service manual and interacting with angry customer by gender

Variables	Odds ratio (95% confidence interval)		
	Rarely	Sometimes	Always
Use of customer service manual (Men)			
Yes	**1.45 (1.22–1.73)**	**1.94 (1.21–3.13)**	**3.16 (1.38–7.23)**
No	1.00 (reference)	**1.73 (1.36–2.20)**	**2.66 (1.82–3.90)**
Use of customer service manual (Women)			
Yes	**1.24 (1.06–1.44)**	**1.99 (1.34–2.97)**	**8.85 (3.96–19.75)**
No	1.00 (reference)	**1.77 (1.41–2.21)**	**2.02 (1.47–2.78)**

Adjusted for covariates including age, monthly household income, education, weekly working hours, job satisfaction, job schedule, job class, occupational classification, **and time spent managing customers**

## Discussion

The overall association of CSM on employees has not been investigated in a nation-wide scale until now. This study indicates the role of CSM in increasing emotional burden on service workers. As shown in Tables 2 and 3, by limiting the free expression of emotion in managing customer needs genders (OR of suppressing emotion with CSM is 1.60 (95% CI 1.39–1.85) for men and 1.55 (95% CI 1.37–1.74) for women), the manual imposes emotional demand on employees which then can lead to psychological problems such as stress or depression (OR of emotional burden is 1.40 for men (95% CI 1.19–1.64) for men and 1.25 (95% CI 1.09–1.44). Interestingly, woman workers seem to be affected more severely than man counterparts by the use of CSM. Although woman employees, when compared to man employees, showed a slightly lower risk of developing stress in presence of CSM at work, they had a higher risk in experiencing depressive events, which is a more serious psychological problem (OR of depressive event with CSM is 1.15 (95% CI 0.52–2,53) for men and 2.22 (95% CI 1.42–3.48) for women). Moreover, synergistic effect between woman workers using CSM and always engaging with demanding customers was distinguished. The same is not true for man workers. (OR 3.16; 95% CI 1.38–7.23 for man who uses CSM and always interact with angry customers and OR 8.85; 95% CI 3.96–19.75 for woman counterparts). These results indicate that CSM harms workers’ mental health and can be more detrimental to woman employees.

The full mechanism between suppressing emotion to display required emotion and mental health is not fully investigated yet. Some studies propose that the energy necessary to regulate emotion severely ills psychological health [20], resulting in the increased risk of mental fatigue [21]. Regulating emotion mandates mental effort and arousal [22]. Continued regulation of emotion can put burden on mental health resulting in mental exhaustion, which can cause emotional burnout [23]. Therefore when workers are constantly exposed to emotional dissonance, psychological resource is depleted, leading to severe mental problems such as anxiety and depression symptoms [24].

In this study, CSM was shown to be associated with suppressing emotion as expected. Since employees are asked to display specific emotion in certain situations, they need to limit their true feelings. CSM eventually advocates emotional dissonance in workers. CSM is also related to emotional burden. Although CSM may have positive effect on psychological health of workers, the overall impact is detrimental. In deciding how CSM harms mental health, emotional dissonance was evaluated. As Indregard et al. illustrated, suppressing emotion showed high correlation with emotional burden in this study [25]. Therefore, it can be said that CSM increases the risk of emotional burden through promoting emotional dissonance in employees. This is an expected result given that emotional dissonance is thought to be related to mental fatigue [21].

The positive association between CSM and emotional burden was present in both man and woman workers. However, the degree of its impact was different between the two groups. In this study emotional burden was measured by a combination of stress and depressive events, with stress assumed as a less serious form of psychological problem. When only considering stress, man workers showed higher correlation than woman counterparts. On the other hand, in the case of depressive event, woman workers showed higher correlation. In fact, for man employees there seemed to be no significant relationship between CSM and depressive events. Although the correlation between CSM and emotional burden is stronger in man workers, the damage on mental health is minor as CSM only increases the risk of stress rather than more serious psychological problems. Woman workers who use CSM have more detrimental outcome as the use of the manual is highly correlated with depressive events. Therefore CSM imposes on woman workers the greater emotional burden.

The discrepancy between the genders is also highlighted when employees are dealing with demanding customers. When workers need to please angry or complaining customers, they are required to exert more effort in regulating emotion which then depletes psychological resources more quickly. As expected, there was an ascending trend between the time spent engaging demanding customers and emotional burden in both sexes. Also in line with the results from this study, there was an increase in the risk of emotional burden in the presence of CSM regardless of time spent with angry customers. Similar trend was reported in other studies where frequency of customer verbal abuse was linked with emotional exhaustion [26]. When customers are angry, they are more likely to exert verbal aggression on employees which in turn leads to greater emotional dissonance and psychological problems. What is surprising is the dramatic increase in risk of emotional burden in woman employees when they are required to manage demanding customers most of work hours while using CSM. Unlike their man counterparts, there was a synergistic effect between the manual and always tending to demanding customers. The combined effect of CSM and dealing with angry customers is much greater than the sum of respective effects.

The different impact CSM can have on genders is not yet been studied previously. One of the possible explanations for this discrepancy can be the difference in job authority. According to The Economist, women hold 10.5% of managerial positions in South Korea and only 2.4% of company boards position [27]. Due to the current social structure, greater portion of woman workers have low job control than man counterparts. In various studies, low job authority is found to be linked with emotional exhaustion [28] and psychological diseases [29]. Having low job control is also associated with suicidal ideation [30]. Moreover, employees with low job authority is more likely to experience mental fatigues when they are burdened with high emotional demand [14]. Therefore, since CSM puts emotional burden on woman workers who are more likely to have low job control, its effect on woman employees is more detrimental. Also when emotional burden becomes greater due to demanding customers, women who have lower job authority deplete emotional resources much more quickly.

### Limitation

This study has a number of limitations to be addressed. First, it is a cross sectional study based on survey questions. So a definite causal relationship between CSM and emotional burden cannot be investigated. In other words, CSM may have been implemented in places where emotional burden is already high to relieve the stress. However, even if that is the case, CSM is not doing a sufficient job to alleviate emotional burden according to this study. Furthermore, dose response manner in current study support our interpretation that CSM can aggravate emotional burden. Second, in the study, emotional burden was defined as experiencing stress at work or having depressive or anxiety symptoms in the past 12 months. Although these two psychological problems constitute a large portion of mental fatigue, it is not a direct measure of emotional burden. Moreover, stress and depressive events are determined by self-assessment and therefore may not be an accurate representation of actual psychological problems.

## Conclusion

It is evident from the study that customer service manual, though it may benefit customers, can put severe emotional burden on employees. The manual promotes emotional dissonance in workers by limiting the freedom of expression. Although both genders are at a danger of psychological problems, woman employees seem to be affected by CSM more severely. This can be due to the current social structure where women are involved in jobs with low job control. Although some could argue that CSM has been implemented only where emotional burden is high to alleviate the burden. However, even if that is the case, CSM is insufficient in relieving emotional burden and actually increasing the burden as seen from the interaction effect. Therefore our current study push researchers and managers to review purpose of CSM again and recommend changes in CSM is necessary to protect workers, especially woman employees, from the danger of emotional burnout.

## Abbreviations

CSM: Customer Service Manual
KWCS: Korean Working Condition Survey
